# A Case of Hydrophobia, with a Brief General Review of the Disease

**Published:** 1879-08

**Authors:** N. Senn

**Affiliations:** Member of the Committee on Surgery


### ARTICLE III.

### A CASE OF HYDROPHOBIA, WITH A BRIEF GENERAL REVIEW OF THE DISEASE. By N. SENN, M.D., Member of the Committee on Surgery. Read before the Rock River Med. Society, May 21st, 1879.

On April 10th, I was called to see Mr. P. S., residing in this city. He was 30 years of age, Norwegian, and a sailor by occupation. He was a man of temperate habits, and with the exception of occasional attacks of indigestion, had always enjoyed good health. On my arrival at the house, I found the patient sitting in bed and elicited the following history: He had felt well and continued to work until the evening previous, when he felt a vague pain through his left ear, with a general feeling of malaise, and began to experience considerable difficulty in swallowing fluids. He had spent a sleepless night, the difficulty in swallowing increasing rapidly. He was very excitable and answered my questions hurriedly. Besides difficulty in swallowing, he complained of a sense of tension in the upper part of the nasal fossa. Pulse 96, full but soft; temp. 38.4°; pupils normal in size, responded promptly to light. I handed him a glass of water, the sight of which appeared to excite severe spasms of the laryngeal muscles and tremors of the extremities. An attempt to drink increased the paroxysms, and had to be abandoned after repeated fruitless efforts. Mistrusting the character of the disease, I cautiously inquired to ascertain the possibility of infection by a rabid animal, and ascertained that a dog belonging to the family had sickened and died seven weeks before; that a neighbor's dog was now suffering in a similar manner, and that the patient had administered remedies to the sick dogs at different times, not being aware of the nature of their disease. The continued gagging and efforts at swallowing on the part of the brutes led the people and the veterinary surgeon to believe that a bone had lodged in the throat. At a subsequent visit, I learned that the first dog, when he had been sick for two days, licked a small abrasion on the patient's left ear.

Although I had never seen a case of hydrophobia, from the history of the case and the characteristic symptoms, I was firmly convinced that the patient was suffering from this terrible malady, and informed his friends accordingly, cautioning them at the same time in regard to the danger of infection. As light, noise and currents of air appeared to exaggerate the symptoms, I directed the patient to be kept quiet in a darkened room, with a limited number of attendants; I prescribed opium in liberal and frequent doses to produce rest, and small pieces of ice to allay thirst.

April 11, 8 a. m.—Found the patient in a state of uncontrollable restlessness; he had slept none during the night; the attendants were unable to keep him in bed; he would walk and jump from one room into another in a fearful state of excitation. He was rational, but despondent, talking freely of the impending fatal result. His eyes were staring and suffused; pharyngeal and nasal mucous membrane congested, temperature 39°, pulse 100. Although the nature of the disease had been withheld from the patient, he appeared to be cognizant of it, and repeatedly assured those around him that they need not be afraid of him as he would not hurt them. As the sight of fluid invariably increased the spasm, I directed that the fluids should be placed in a cup and taken through a rubber tube; this device seemed to render deglutition more easy. Attempting to drink, the patient would jump forward, stand firmly on the floor, hold his head to one side and convulsively grasp the cup and tube, then make a desperate attempt to swallow which would generally fail, the next two or three efforts were more successful. Solid food was masticated and swallowed readily. Sometimes the fluids that had apparently been swallowed would be forcibly ejected before reaching the stomach. I now directed the following powder to be taken every two hours:

| | |
|---|---|
| Camphora.......................................... | 133 |
| Morphia sulphas.................................. | 02 |

with a liberal allowance of brandy (alcoholic stimulants were swallowed more readily than water).

Several physicians were called in consultation during the day, most of them corroborated the diagnosis and endorsed the treatment.

12 m.—The symptoms were greatly aggravated, the expression of the face indicated an indescribable distress; there was hyperæsthesia of all the senses, the least touch, a current of air, noise, a bright light or any slight disturbance of the patient was sufficient to cause terrible convulsions. The patient never remained in any one place or position longer than a few minutes, and talked in an excited manner, alluding frequently to the intensity of his sufferings. A copious secretion of viscid mucus from the pharynx and mouth was the cause of constant hawking and spitting. It was only by the most powerful efforts of the will that he was able to swallow small quantities of fluid. Spasmodic contractions of the muscles of respiration occasioned a feeling of suffocation, and of pain in the epigastrium.

9 p. m.—Although the medicines have been administered regularly, they have secured no rest. The patient is in a state of constant agitation. Countenance is livid, extremities cold, skin covered with a clammy cold perspiration, pulse 160 and almost imperceptible, delirious, deglutition almost impossible. The patient continued in a state of restlessness, being tormented with almost uninterrupted spasmodic contractions of different parts of the body until midnight, when death, seemingly from exhaustion, came to his relief. Six hours after death I examined the corpse. The whole surface presented a dark blue appearance, rigor mortis was well marked. The abrasion on the left ear, through which infection had undoubtedly taken place, had healed without a scar and the ear presented no other abnormal appearance. This case, the first I had ever seen, and, I believe, the first ever observed in this city, led me to look up the literature of this rare disease, for a report at this meeting.

Hydrophobia, rabies canina, or, as it is sometimes called, lyssa, is a disease peculiar to the canine species of animals — as the dog, wolf, fox, hyena and jackal — in whom it may originate spontaneously, while in other animals and man it only occurs by means of the transmission and inoculation of a specific virus.

It is an acute, infectious disease, characterized by a functional disturbance of the nervous system, unattended by any appreciable constant anatomical lesions.

The disease was known to the ancients, and we find frequent allusions to it in the writings of poets and historians, as well as in medical works. Aristotle refers to it in his great work, "Historia Animalium." Galen declared it to be the worst of all diseases. Plutarch regarded it as a new disease, which took its origin during the time of the Asklepiades. Coelius Aurelianus, a contemporary of Augustus, wrote the first book on this disease. It has been described by Celsus, Dioscorides, Plinius, Soranus, Paul of Ægina, Aracteus Cappadocius, Ætius, Rhazes and Serapius. Among the later investigators to whom we are indebted for a more accurate knowledge of this disease, we may mention prominently Maynell and Youatt.

In the human subject the disease never originates *de novo*, but is always the result of inoculation with a specific poison. Infection, in the majority of cases, is due to a bite of a rabid animal, usually a dog, although it may take place by absorption of the virus, when brought in contact with an abraded surface, as was the case in the above instance. Large wounds, as a rule, are less dangerous than small ones, from the fact that in the former the virus is more likely to be washed out by the flow of blood. The specific infecting principle of hydrophobia is contained in the saliva and foam of the infected animal; also in the blood and salivary glands, and perhaps in other tissues of the body. Of its physical properties we are at present ignorant. The virus is reproduced in the infected system, and is distinguished from other poisons by the fact that it remains latent in the organism for weeks or months, without producing any morbid symptoms. The probabilities of contracting the disease after being bitten by an animal known to be rabid, are about equal to those of infection from other causes. According to the statistics of Tardieu and others, about 47 per cent. of those bitten die of hydrophobia. If we include bites of dogs suspected of being rabid, the proportion is even more favorable, only 8 per cent. terminating fatally. It is very doubtful if any reliable cases are on record in which human beings suffering with the disease have communicated it to others by their bite; the possibility of infection however, cannot be denied. It has been asserted by some that the disease is simply a nervous affection, induced by anxiety and apprehension of the dreadful disease, and consequently that it is not produced by a distinct virus. This is contradicted by the fact that the disease has been observed in children two years of age, whose imagination cannot be wrought on by a fear of the disease. One of the physicians who visited my case, sought to explain the symptoms on these grounds, but the patient was not aware of the character of the disease affecting the dogs until the symptoms had fully developed in himself. The frequency of hydrophobia in man depends on the degree of prevalence among animals. In France, in six years, from 1853 to 1858 inclusive, there were 107 cases in a population of 36,000,000, or one annually for every 2,000,000. In the city of New York, during six years, from 1866 to 1871 inclusive, with a population of 1,000,000, there were 22 cases, or an average of $3\frac{2}{3}$ per annum. In Prussia there occur annually, on an average, 71 deaths from rabies. In Austria, on an average, 58 deaths take place annually from the same cause.

*Period of Incubation.*—By the period of incubation, we understand the period of time which elapses from the time the virus is brought in contact with a wounded or abraded surface until the first symptoms of disease manifest themselves. It is the time during which the poison remains latent in the system or at the point of introduction. It varies greatly in different cases. According to Boudin, in 147 cases it lasted:

1 month in 26 cases.
1 to 3 months " 96 "

3 to 6 months in 19 cases.
6 to 12 " " 9 "

In the majority of cases it terminates at the end of the second month. The variation in the length of time the poison remains dormant, has not as yet been sufficiently explained. The popular opinion, shared to a certain extent by some of the older writers, that the disease may manifest itself even after several years from the time the poison was introduced, is fallacious, and has given rise to a great deal of unnecessary mental anxiety. If no symptoms manifest themselves after a lapse of six months from the time supposed inoculation took place, the person may be pronounced practically safe. During this stage there are no symptoms indicating that so appalling a malady is in process of development. The wound heals kindly, even after the application of caustics.

*Symptoms.*—When the disease begins to manifest itself, the cicatrix often becomes swollen and reddened, and sensations of prickling, boring or tearing pains are experienced in the wounded parts or in adjacent tissues. In my case the patient complained of pain in the ear and the same side of the face several hours before any other symptoms had shown themselves. The characteristic signs of the disease come in rapid succession. The patient complains of languor, and a feeling of stiffness about the neck, extending to the jaw and base of the tongue. He is affected with great restlessness, and an indescribable anxiety. Thirst is extreme, but, owing to the distress occasioned by an attempt to swallow fluids, there is an aversion to liquids. Solid food is generally swallowed without much difficulty. The mouth at first is dry, and the salivary secretion diminished. In some cases vomiting is an early symptom ; the bowels are generally constipated and the urine scanty. The special senses are morbidly sensitive ; light, sound and other external impressions produce an exaggeration of symptoms. Sexual desire, more particularly in the male sex, is increased during the early accessions of the disease. At the onset the pulse is somewhat increased in frequency, and the temperature higher than normal. The patient is inclined to talk almost constantly, frequently changing the subject of conversation. The nervous excitation increases rapidly in severity, until convulsions of a paroxysmal character occur; these are usually preceded by spasmodic constrictions of the pharynx. Instead of a dryness in the mouth, a copious viscid secretion now distresses the patient, who, on account of difficulty in swallowing it, ejects it forcibly in every direction. Although mental illusions and hallucinations occur during the convulsive attacks, during the intervals the mental faculties are for the most part retained. The continued convulsive movements, the loss of sleep, and the inability to take nourishment, rapidly exhaust the strength of the patient. The pulse becomes more rapid, the extremities cold, the skin livid, the eyes suffused, the convulsions follow at shorter intervals, although perhaps less in severity, a cold, clammy perspiration makes its appearance, symptoms of exhaustion become more and more apparent until death closes the scene.

*Diagnosis.*—The only diseases which might be mistaken for hydrophobia are hysteria and tetanus. The use of the thermometer will exclude hysteria with certainty, as the temperature in this disease when uncomplicated, is normal, and in hydrophobia is always increased. Tetanus in many respects resembles hydrophobia. In both diseases the reflex function of the spinal cord is increased; both prove ordinarily fatal in a few days; both present violent paroxysmal convulsions, increased and excited by external irritating influences. In the essential features they are, however dissimilar. In tetanus deglutition may be difficult, in hydrophobia it is next to impossible. In the former, respiration may be more difficult; in the latter the spasms of the respiratory muscles are almost as conspicuous as the spasms of deglutition. In hydrophobia a viscid mucus accumulates in the mouth provoking frequent spitting; constant thirst is a distressing feature; the patient is more irritable and restless; there is tremor of the muscles and the spasms are more often clonic instead of tonic. If, in addition to all this, we are able to ascertain the infliction of a bite from a rabid animal, a few weeks or months previous to the attack, the diagnosis remains no longer doubtful.

*Prognosis.*—According to our best informed and most reliable authorities, the disease proves fatal without exception. Death takes place usually on the second or third day. The patient may die in the midst of a paroxysm from apoplexy or asphyxia, but most frequently it takes place from exhaustion after a state of collapse. It is true cases have been reported where it has been claimed that recovery occurred, but the possibility of an error in diagnosis in these cases must always be taken into consideration. Faber collected the histories of 19 cases where recovery is said to have taken place. One case is reported by Hanauld, as cured by means of the cold douche continued until syncope was produced. Ottman and Falkner each report a case treated by mercurial inunction. Sauter reports two cases which yielded to the administration of belladonna, and Dr. Watson claims for the hypodermic injection of woorara the same beneficial results. As regards the prognosis of bites by rabid animals, this is influenced by the locality of the wound, and the kind of animal inflicting it. A bite of a rabid wolf or fox is more dangerous than that of a dog. According to Baulley, of wounds of the face, 90 per cent. proved fatal; of the lower extremities, 28 per cent., and of the upper extremities, 20 per cent. When immediate cauterization is practiced, 33 per cent. of those bitten by rabid animals die of rabies, while in cases where no such precaution is practiced, 83 per cent. prove fatal.

*Pathological Anatomy.*—Early and well marked rigor mortis takes place after death. The blood is fluid and dark, its coagulability being greatly diminished. Although the symptoms during life point directly to the nervous centers, and more particularly to the spinal cord as the seat of lesion, no distinct and constant pathological changes have been observed.

*Pathology.*—The disease undoubtedly depends upon the introduction into the circulation of a specific poison, which is reproduced in the body. As yet the location of the poison during the period of incubation has not been determined. The greater number of observers believe that it remains latent in the cicatrix until active symptoms begin to develop themselves, while some are of the opinion that it is immediately introduced into the circulation, and after it has reproduced itself to a certain extent it produces the characteristic symptoms. I am inclined to adopt the latter theory as correct. If the former were correct, the poison might remain innocuous in the cicatrix for an indefinite period of time. The one way to explain the variable length of the period of incubation, is to assume that the reproduction of the poison is not carried on with the same rapidity in different individuals. The same discrepancy in this respect is observed in some of the other infectious diseases. The poison, when fully developed, seems to expend all its force in disturbing the reflex functions of the cord.

*Treatment.*—This divides itself into the prophylactic, palliative and curative. The prophylactic treatment consists in removing or destroying the virus in the wound. For this purpose the practice of Cornelius Celsus has been little, if any, improved upon. He says: "The virus should be withdrawn by means of a dry cupping-glass, and the actual cautery applied to the wound; if this be impossible, then other caustic agents should be applied and the parts should be bled." The bleeding of the wound should be encouraged. Besides the actual cautery, various chemical substances have been used at different times, as caustic potassa, nitrate of silver, antimony, ammonia, mineral acids, for the purpose of destroying the poison in the wound. Cauterization has also been practiced after the wound had completely healed, and, according to Harder, Hicks and Griesly, has been found of benefit even after the disease had made its appearance. Virchow recommends Vienna paste as the most reliable caustic. Excision of the wound has not been found more effective than cauterization, and as it is more difficult to perform, it should not be resorted to as a rule. If the punctured wounds are deep, it may be necessary to enlarge them to facilitate the application of the caustic. No internal remedies have been found of avail to neutralize the poison during the period of incubation, and thereby preventing the development of the disease. Of the innumerable popular and secret remedies, none have answered this expectation. After the disease has become fully established, our efforts must be directed mainly towards palliation. The present state of medical science has no claim to a curative remedy. Venesection, transfusion, intra-venous injection of warm water, mercurials, belladonna, arsenic, hydrocyanic and carbolic acids, the subcutaneous injection of woorara, atropia and quinine have all been tried for this purpose, but in vain. As palliatives to allay spasm, relieve pain and procure rest, opium, chloroform and chloral may be used either separately or in combination. When the chemist or microscopist has isolated the specific poisonous principle, then the therapeutist will go to work intelligently and find some agent that will destroy it or neutralize its effects, and thus eradicate from our death-list the most appalling and fatal of all diseases.

LITERATURE.

Billroth's Surgical Pathology.
Flint's Practice of Medicine.
Reynold's System of Medicine.
Ziemssen's Cyclopædia of Practice of Medicine.
Emmert's Chirurgie.
Walther's System der Chirurgie.
Billroths & Pitha's Allg. u Specielle Chirurgie.
Gross' System of Surgery.
*American Journal Med. Sciences* (Dr. Watson).
Erichsen's Surgery.

